# Epidemic intelligence in Europe: a user needs perspective to foster innovation in digital health surveillance

**DOI:** 10.1186/s12889-024-18466-1

**Published:** 2024-04-06

**Authors:** Fanny Bouyer, Oumy Thiongane, Alexandre Hobeika, Elena Arsevska, Aurélie Binot, Déborah Corrèges, Timothée Dub, Henna Mäkelä, Esther van Kleef, Ferran Jori, Renaud Lancelot, Alize Mercier, Francesca Fagandini, Sarah Valentin, Wim Van Bortel, Claire Ruault

**Affiliations:** 1 Groupe d’Expérimentation et de Recherche: Développement et Actions Locales (GERDAL), Angers, France; 2 Joint Research Unit Animal, Health, Territories, Risks, Ecosystems (UMR ASTRE), French Agricultural Research Centre for International Development (CIRAD), National Research Institute for Agriculture, Food and Environment (INRAE), Montpellier, France; 3 UMR MOISA, French Agricultural Research Centre for International Development (CIRAD), 34398 Montpellier, France; 4 MOISA, University Montpellier, CIHEAM-IAMM, CIRAD, INRAE, Institut Agro, Montpellier, France; 5 Joint Research Unit EPIdemiological On Animal and Zoonotic Diseases (UMR EPIA), National School of Veterinary Services (VetAgro Sup), National Research Institute for Agriculture, Food and Environment (INRAE), Marcy L’Etoile, France; 6 Department of Health Security, Finish Institute for Health and Welfare, Helsinki, Finland; 7 Joint Research Unit Land, Remote Sensing and Spatial Information (UMR TETIS), French Agricultural Research Centre for International Development (CIRAD), Montpellier, France; 8 Institute of Tropical Medicine, Department of Biomedical Sciences, Outbreak Research Team, Antwerp, Belgium; 9 Institute of Tropical Medicine, Department of Biomedical Sciences, Unit of Entomology, Antwerp, Belgium

**Keywords:** Zoonotic diseases, One health, Digital tools, Event-based surveillance, Big data, Sociology of innovation, Sociology of work, Co-design, Co-creation, Co-conception

## Abstract

**Background:**

European epidemic intelligence (EI) systems receive vast amounts of information and data on disease outbreaks and potential health threats. The quantity and variety of available data sources for EI, as well as the available methods to manage and analyse these data sources, are constantly increasing. Our aim was to identify the difficulties encountered in this context and which innovations, according to EI practitioners, could improve the detection, monitoring and analysis of disease outbreaks and the emergence of new pathogens.

**Methods:**

We conducted a qualitative study to identify the need for innovation expressed by 33 EI practitioners of national public health and animal health agencies in five European countries and at the European Centre for Disease Prevention and Control (ECDC). We adopted a stepwise approach to identify the EI stakeholders, to understand the problems they faced concerning their EI activities, and to validate and further define with practitioners the problems to address and the most adapted solutions to their work conditions. We characterized their EI activities, professional logics, and desired changes in their activities using NvivoⓇ software.

**Results:**

Our analysis highlights that EI practitioners wished to collectively review their EI strategy to enhance their preparedness for emerging infectious diseases, adapt their routines to manage an increasing amount of data and have methodological support for cross-sectoral analysis. Practitioners were in demand of timely, validated and standardized data acquisition processes by text mining of various sources; better validated dataflows respecting the data protection rules; and more interoperable data with homogeneous quality levels and standardized covariate sets for epidemiological assessments of national EI. The set of solutions identified to facilitate risk detection and risk assessment included visualization, text mining, and predefined analytical tools combined with methodological guidance. Practitioners also highlighted their preference for partial rather than full automation of analyses to maintain control over the data and inputs and to adapt parameters to versatile objectives and characteristics.

**Conclusions:**

The study showed that the set of solutions needed by practitioners had to be based on holistic and integrated approaches for monitoring zoonosis and antimicrobial resistance and on harmonization between agencies and sectors while maintaining flexibility in the choice of tools and methods. The technical requirements should be defined in detail by iterative exchanges with EI practitioners and decision-makers.

**Supplementary Information:**

The online version contains supplementary material available at 10.1186/s12889-024-18466-1.

## Introduction

The threat of (re)emerging infectious diseases (EIDs) has increased due to global changes, including climate change, and the increasing global movement of people and goods. Recent pandemics have highlighted the vulnerability of traditional indicator-based surveillance (IBS) systems for the early detection, monitoring and assessment of EIDs [1]. The detection of and early responses to emergencies will continue to challenge European disease surveillance systems. More than 60% of EIDs are zoonoses, and their incidence has significantly increased over time [2], stressing the relevance of a One Health approach [3]. Its implementation faces many challenges from the design of collaborations between sectors up to the integration of intersectoral data for shared risk assessments, hence placing an additional burden on public health (PH) and animal health (AH) epidemic intelligence (EI) services [4, 5].

To complement traditional IBS based on mandatory disease notifications, sentinel surveillance, syndromic surveillance, and other structured alerts [6], many national surveillance systems have established event-based surveillance (EBS) activities and services for EI. The EI encompasses all activities related to early identification, verification, analysis, assessment, and investigation of health threats and integrates both IBS and EBS activities. The IBS uses systematically collected surveillance data, whereas the EBS uses unofficial, unverified, unstructured data from multiple sources. The importance and high value of EBS is stated by the International Health Regulation (IHR) [1, 7], which established a legal framework for EI aiming at early detection, reporting and response to EID outbreaks. The three pillars of internet-based EBS are disease and syndromic surveillance of the news or based on participatory tools, social media analysis (Twitter, Facebook, etc.), and aggregated internet search trends (e.g., Google search trends) [8, 9]. The availability of new information sources and methods to explore large volumes of data leads to new challenges in managing, analysing and interpreting these data flows [10].

The European Commission (EC) supports the concept of an innovation process to respond to EID and the growing threat of antimicrobial resistance (AMR). This process should be built on the principle of openness (open science, open innovation and open to the world of societal challenge) and aims to develop a European market of digital tools in the health sector that could promote improved and sustainable digital practices for EI [11].

A recent quantitative description of the EI activities of national agencies [12] brought some new ideas about the data needs of national PH and AH agencies. A complementary qualitative assessment can provide insights into practical difficulties, the underlying problems they want to solve, and the professional expectations of EI practitioners, elements that are necessary to design strategies and tools for changes in practices.

The aim of our study was to describe the activities of European EI practitioners and their needs for innovation within the framework of their professional EI activities. We called “EI practitioners” the epidemiologists of national and international PH and AH agencies with a mandate of EID detection, regardless of the relative importance of strategic choices based on IBS and EBS in terms of sources, tools and methods for early detection. In addition, we aimed to co-design possible adaptations of new tools and services for EI. This approach is based on the idea that innovation, in the sense of creating and implementing sustainable changes in practices in a professional group [13, 14], is a process grounded in the operational objectives and action logics of practitioners: *"The problems are not given by the situations but by those who experience them"* [15]. To be implemented properly, the changes must be considered as answers to practical questions posed by practitioners in the course of their work. We provide the first analysis of innovation needs in terms of digital health surveillance in the area of EI related to EIDs and unknown diseases (“Disease X” identified by the WHO (World Health Organization)), considering both the PH and AH sectors in Europe.

## Materials and methods

### Study design and data collection

We used a socio-technical approach [15, 16] to identify both i) how EI activities and services are organized at the national level and at the ECDC and ii) the actors' practices and their professional network. We aimed to understand the difficulties they encountered, the problems they wanted to solve, and to identify possible solutions. Additionally, we summarized and validated this initial assessment and collectively prioritized the needs and paths of solutions during workshops with the interviewed practitioners, researchers and engineers.

We targeted epidemiologists who have an official mandate for the early detection and monitoring of infectious threats according to the following EID types: AMR, food and water-borne diseases (FWBD), vector-borne diseases (VBD), respiratory diseases, and unknown diseases. We involved these epidemiologists and one entomologist as gatekeepers and potential users of new digital EI tools and services, who were subsequently called EI practitioners.

**Fig. 1:**
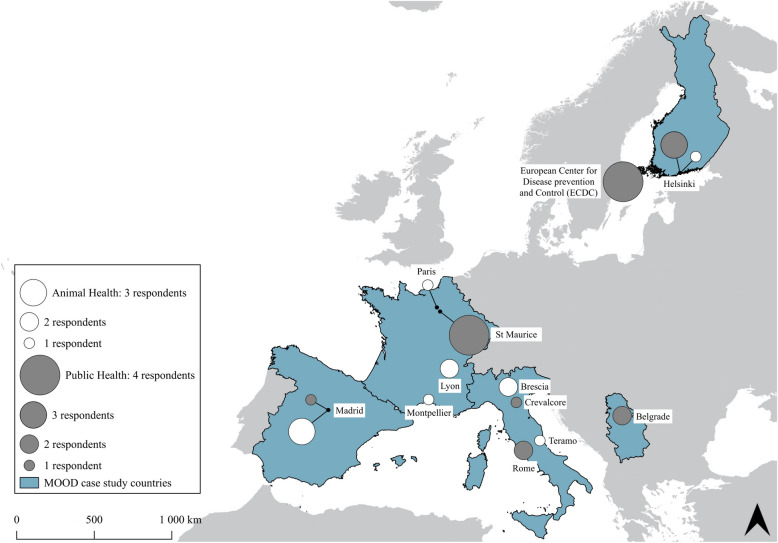
Model countries and number of interviewees by sector and location involved in the 28 in-depth interviews of the user needs assessment

The study was carried out in five national European PH and AH agencies and a supranational agency represented by the ECDC. The five countries were chosen for diverse health information systems (HISs) and ecological situations (North, South, East and West continental Europe) (Fig. 1): they became model countries for the co-creation of numeric tools in the framework of the H2020 MOOD project (MOnitoring Outbreak events for Disease surveillance in a data science context; https://cordis.europa.eu/project/id/874850).

**Fig. 2:**
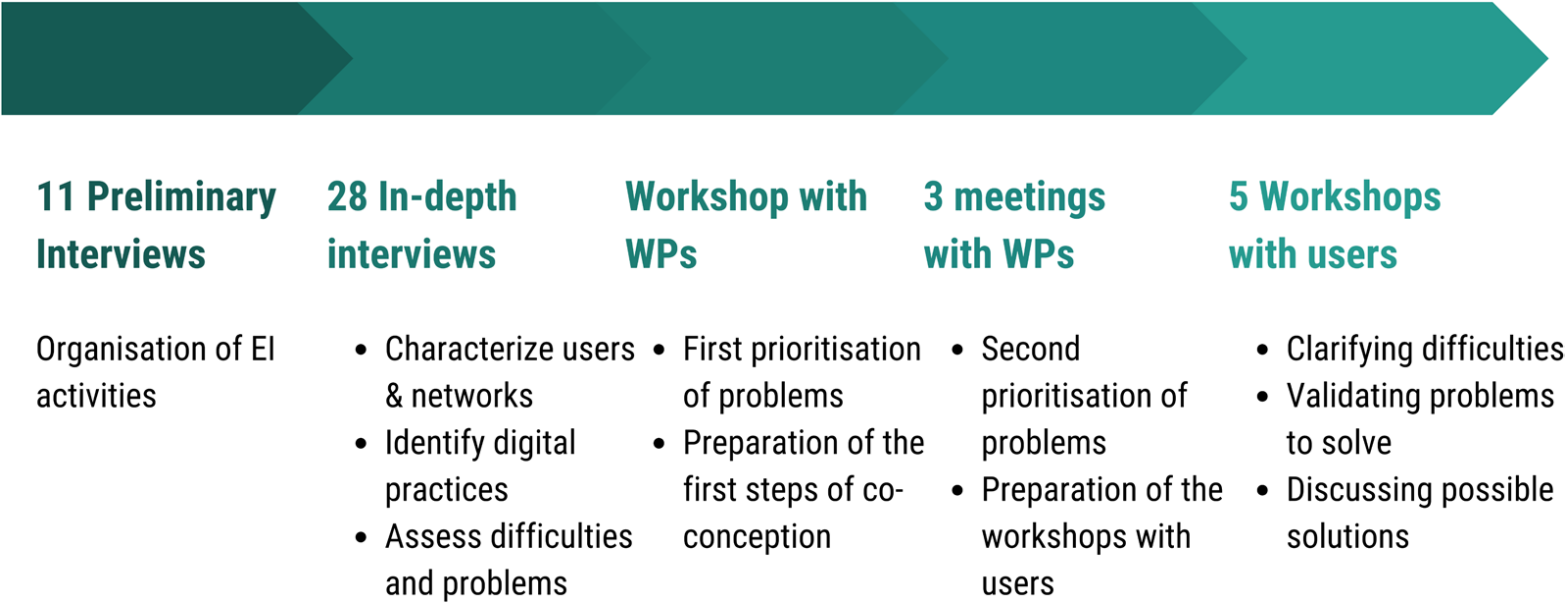
Process for initial user needs assessment and prioritization implemented by the MOOD consortium in 2020. WP: Work groups of the consortium

In accordance with the principles of comprehensive sociology and research action approach [15, 16], several loops of interactions were implemented based on a stepwise method (Fig. 2) using different tools, including questionnaires, interview guides, and a digital tool for the exchange of ideas (Klaxoon ®):

- Step 1: Describe the EI activities in each country and sector and identify EI practitioners to interview.
- We interviewed one key informant, or one group of informants per country and sector, and one key informant for the ECDC (Jan.-Feb. 2020). The informants were epidemiologists working in a dedicated EI team or having a good overview of the surveillance of the preselected EID types (proposed by the agencies). The telephone interviews were conducted by epidemiologists using a semi-structured questionnaire (Supplementary file 1).
- Step 2: Understand the professional practices related to the management of epidemiological data and identify the problems to be solved and expectations in terms of the types of solutions.
- We conducted 28 in-depth interviews (duration about 1h30) by telephone using semi-structured interview guides with 12 key informants involved in Step 1 and 16 additional epidemiologists (with various backgrounds) or entomologists performing surveillance and risk assessment on the selected list of diseases relevant for Europe (Table 1; Fig. 1). These 16 additional interviewees were identified by sociologists on the basis of the preliminary interviews. We ensured a gender balance among the interviewees (Table 2). The interviews focused on position, mandate, activities, practices of data management, difficulties encountered, priority problems, and suggested paths of solution. The interviews were recorded upon informed consent of the interviewees (Mar.-Nov. 2020).
- Step 3: Interact with researchers from different disciplines, i.e., epidemiology, computer science, modelling and sociology, to categorize and prioritize the problems that could be solved through solutions based on new open-source tools and data. The interaction was implemented through a workshop by teleconference with PowerPoint documents;
- Step 4: Interact a second time with the interviewees together with researchers in epidemiology, computer science, modelling and social sciences, based on three workshops by teleconference with PowerPoint and Excel documents;
- Step 5: Collectively validate the problems to solve and detail the problems and paths of the solutions. Five workshops were implemented by teleconference with the interviewees of Step 2 plus five practitioners (added by snowballing) and researchers (at least one from each discipline). The Klaxoon® boards were used as supportive tools (Dec. 2020).

**Table 1:** Summary of the EI activities of the interviewees

Country	Sector	EI activities of the interviewees
Europe	PH	Modelling of trends and non-pharmaceutical interventions to inform risk assessment (IBS)
		Detection and assessment of threats from infectious diseases (EIDs) including disease X (EI)
		Detection and assessment of threats from EIDs including disease X (EI)
		Detection and assessment of threats from EIDs including disease X (EI)
Italy	PH	Monitoring and risk assessment in entomology (IBS)
		Surveillance of enteric pathogens (IBS)
		Detection and risk assessment of EIDs threats (national EI, and IBS for VBD)
	AH	Surveillance in animal health and food safety (IBS mainly)
		Monitoring program about bacterial diseases, AMR, biosecurity and animal welfare (IBS)
		Monitoring of West Nile disease virus and Usutu virus (IBS)
France	PH	Epidemiologic surveillance of tropical diseases: detection and risk assessment of introduction (IBS)
		Management of surveillance systems for arboviroses (IBS)
		Management of surveillance systems for AMR (IBS)
		Surveillance of hepatitis A, E, tularaemia, and coordination of tick-borne diseases (TBD) surveillance (IBS)
	AH	Coordination of the IBS groups and the EI group of the platform (EBS and IBS)
		Editor of the national epidemiological bulletin (AH and food security); international EI (EBS)
		Detection and assessment of threats from EIDs including disease X (international EI)
		Detection of OH threats (low signals monitoring) (EBS)
Finland	PH	Preparedness, response, risk assessment (IBS)
		Infectious disease consultant dedicated to the Hotline (EBS)
		Investigations on bacteria and food-borne pathogens (IBS)
	AH	Modelling risk assessment for animal diseases (IBS)
Serbia	PH	Surveillance, detection and reporting (IBS)
		Detection and assessment of VBD (IBS)
Spain	AH	Surveillance, detection, management and elaboration of guidelines (IBS)
		Management and implementation of european law in disease surveillance and VH (IBS)
		Implementation of program and reporting on zoonosis and AMR for EC (IBS)
	PH	Coordination and management of information system (IBS)

**Table 2:** Age, gender and seniority of the interviewees

Gender	Number	Average age	Average seniority
Female	14	43.8	6.9
Male	14	48.7	11.1
Total	28	46.4	9.0

An additional series of dedicated meetings about the needs related to highly pathogenic avian influenza (HPAI) were organized during the first trimester of 2021 in response to recent requests expressed by the AH authorities of France concerning modelling outputs to support decision-making.

### Data analysis

The analysis of the interviews performed in Steps 1–2 (Fig. 2) was performed in the following four thematic areas, corresponding to our research objectives: professional mandate and objectives; surveillance practices and professional network; difficulties and problems to be solved; and useful data and tools. We created tables to highlight the different types of difficulties and problems to be addressed as expressed by the practitioners and identified points of discussion as criteria of vigilance or paths of solution mentioned by the interviewees. We then performed a thematic analysis on all the collected information with Nvivo® 12 software. We used 28 preselected nodes that corresponded to central subthemes identified by each segment of discussion (Supplementary file 2). Each node was annotated, and a memo was generated for each node. The interviews, annotations, and memos were transversally analysed. We cross-checked the nodes that were linked and made a general comparison between interviews related to each node.

Regarding the workshops (first without EI practitioners, then with them; steps 3, 4 and 5; Fig. 2), the main topics discussed were summarized in a report, and the causes of difficulties, statements of the practitioners, and problems to solve were further defined, complemented or reformulated in tables. To characterize the diversity of the difficulties and problems, categories were built in an inductive way (related to the concerned step of the activity and the nature of the difficulties) (Supplementary files 2 and 4).

## Results

The results presented below are the outputs specified and validated after step 5 (Fig. 2).

### Description of EI systems and activities

Fifteen epidemiologists from the PH and AH agencies in five countries and the ECDC were interviewed during 11 preliminary individual or collective interviews. Among them, 12 were involved in the second round of interviews, and 16 other epidemiologists from these networks were selected according to their position for the 28 semi-structured interviews (Fig. 1). Five additional practitioners participated in the workshops and HPAI meetings. Table 1 presents the EI activities executed by the 28 interviewees (14 women and 14 men): 61% of them worked in a PH agency, and 39% worked in an AH institution. The average age of the interviewees was 46.6 years, and the seniority was 9 years, with higher average ages and seniority for men than for women (Table 2).

The organization of EI activities (Supplementary file 3) varied in terms of mandate, centralization, human resources and data sources from one institution to another and between EI teams. EID and biothreat detection and surveillance were the core mandates, and the mandate concerning bioterrorism was sometimes delegated to a specific centre. AMR was sometimes addressed in different EI networks than the central network (not addressed by the platform ESA (French platform for epidemiological surveillance in animal health) and addressed by different agencies (France, PH)). At the national level, IBS, based on mandatory notification of disease, sentinel and syndromic surveillance, played prominent roles in EI.

Most of the human resources of national agencies were dedicated to national IBS, and very few were dedicated to national EBS, and even fewer were dedicated to international EBS. The largest national group dedicated to EI (Italy PH) consisted of 20 officers mainly working on IBS but covering national EBS activities through a rotation of duties. In Spain, international EBS was specifically externalized to a public–private partner. When international EBS was not part of the mandate of the team, it was often implemented in a non-formal manner, according to the sanitary situation, in addition to the IBS activities (France PH, Serbia PH, Italy AH).

National health information systems (HISs) were mainly based on heterogeneous non interoperable institution-specific databases, more interoperable dataflows from laboratories and sometimes on shared multilevel repositories from surveillance networks (Spain, Italy). The shared online national platforms (multi-institutions, multilevel and multisectoral) were recent or under development and did not address all the diseases. Some specific challenges were linked to the regionalization of Spain and Italy and related autonomy in the organization of disease surveillance.

The ECDC is an agency offering European countries the outputs of its EI activities or its support to develop the countries’ own EI services. The ECDC, ISS (Istituto Superiore di Sanita) from Italy (for PH) and ESA platform from France (for AH) had dedicated teams implementing EBS and standardized operational procedures for EBS.

The use of international platforms for reporting (TESSy (The European Surveillance System from ECDC), EFSA (European Food Safety Authority), WHO, ADNS (European Commission's Animal Disease Notification System), sharing of events (EWRS (The Early Warning and Response System)/EPIS (Epidemic Intelligence Information System, replaced by EPIPULSE), WHO, WAHIS (World Animal Health Information System)) and accessing international validated data (notifications and website of WHO, ADNS, WOAH, ECDC)) were generalized for the four European countries and Serbia as an ENC (European Neighboring Country).

Multiple sectoral collaborations (between PH, AH and wild fauna stakeholders) were often implemented for disease-specific activities on an ad hoc basis (for food safety, some VBDs and AMR), without formal One Health standard operative procedures (SOP). The formalization of intersectoral collaborations was facilitated in some countries by the institutional integration of AH institutions under the authority of the Ministry of Health, such as Italy (Table 3), through national plans (against AMR, for communication about zoonosis, for the coordination of alerts in Spain) or through agreements between institutions involved in thematic and transversal working groups (France AH).

**Table 3:** Occurrence of difficulties by type of difficulty for each EI step (for a total of 59 difficulties identified in 28 interviews)

Type of difficulties*							
Tasks	Time-consumption	Technical barrier	Quality	Collaboration	Methodology	Timeliness	Total
Collection	7	3	1	0	1	2	14
Processing	4	4	1	0	0	0	9
Data sharing	1	3	0	3	0	0	7
Analysis	3	3	7	0	2	1	16
Reporting	2	0	0	0	0	1	3
Strategy	0	0	0	5	5	0	10
Total	17	13	9	8	8	4	59
% interviewees	61%	46%	32%	29%	29%	14%	
number of countries	4 + ECDC	3	4	4 + ECDC	4 + ECDC	2	

^*^Time consumption: This constraint is linked to the time needed to implement the task; Technical barrier: lack of capacity linked to a tool, data or any technical aspect; Quality: difficulties linked to the insufficient value of the output/result of the activity (as expected by the stakeholder); Collaboration: difficulties linked to the insufficient or absence of work relations between different agencies or stakeholders involved in surveillance activities; Methodology: difficulties linked to a lack of know-how or consensus concerning methods and tools; Timeliness: in a broad meaning, the difficulties linked to the capacity to address the task at the accurate moment (according to the user’s point of view)

### Implementation of EBS in the model countries

**Table:** 

The EI team of the ECDC was the only centralized team with a rotation of EI officers and a wide network of disease specialists in situ for the daily round table. They also used various EI data sources: official sources at multiple scales on top of their in-house platforms and databases and many informal sources, such as aggregators, participatory surveillance systems, blogs and social media. The Italian EI group (rotation of 20 agents for national EBS and two for international monitoring) was decentralized and used only two EBS tools for national EBS and one additional platform for international monitoring (EIOS, epidemic intelligence from open sources initiative, led by the WHO). The focus on only two tools was possible due to the efficiency of the Italian language as a filter to collect national information and the strong technical support of the European Commission Joint Research Centre. The French AH platform of epidemiological surveillance also had a decentralized group and used a limited number of EI tools, such as the Italian PH EI group. The three dedicated groups used SOPs for EBS. ECDC plays a prominent role in producing and sharing new SOPs. In Spain (AH), national and international EBS was implemented without SOP by a network of officers of the ministry and epidemiologists from a private‒public company to complete IBS (ADIS (EU Animal Diseases Information System) and national databases) for some selected transboundary animal diseases by periodical monitoring of the situation in ENC from various Web sources (aggregators (HealthMap, ProMED), national and international public media, official websites (WOAH sites, WHO, etc.). Specific EBS dataflows based on the notifications of medical practitioners were implemented at the national level: a hotline for healthcare professionals to notify suspicions of EID in Finland, the system RYMY to notify FWBD events (Finland), and an online tool e-SIN to notify healthcare-related infections (France). These data sources were confidential, and some tools were not digital (such as hotlines). In Serbia, systematic media monitoring concerned rumours and PH communication.	

### Main difficulties encountered by EI practitioners

We collected in the interviews 59 difficulties, defined as interviewees’ statements related to tasks seen as “difficult to implement” or corresponding to unfulfilled expectations. We grouped them into six categories (Table 3): time-consuming tasks (17/59 difficulties, mentioned by 61% of the interviewees), technical barriers (13/59, mentioned by 46% of the interviewees), lack of quality (9/59, mentioned by 32% of the interviewees), lack of collaboration (8/59), methodological issues (8/59) and lack of timeliness (4/59, mentioned by 14% of the interviewees). The most frequent difficulties in the interviews were related to the following steps of the EI activities: data collection (14/59 difficulties, mentioned by 50% of the interviewees), data processing (9/59, mentioned by 32% of the interviewees), analysis (15/59, mentioned by 54% of the interviewees), and the strategy to organize surveillance (10/59, mentioned by 36% of the interviewees) (Table 3). Their first mandate was to produce consolidated data, but they were also in charge of producing risk assessments. Many EI practitioners expressed being overwhelmed by the time-consuming data collection, processing, data sharing and reporting activities (14/59, mentioned by 50% of the interviewees), which left them little time for the actual data analysis and interpretation. Many difficulties concerned the analysis, because of the poor quality or lack of data, a lack of dedicated personnel with sufficient training, a lack of coordination to review strategic issues, and a lack of knowledge, know-how and tools for implementing the One Health approach.

Table based on data from Supplementary file 4.

#### Data collection

The difficulty of accessing good-quality data in a timely manner represented a major constraint for national surveillance, early warning, and long-term international monitoring. The COVID-19 crisis (coronavirus disease 2019) has highlighted the limits of the EI system.

*“Now, after the COVID-19 crisis […] we understood that [most of] the failures that we had in some areas of the emergency response […] were due to the poor data availability.”* (Italy P2 PH)

Access to IBS and EBS data was divided into several issues: time-consuming constraints to obtain complete epidemiological information, such as manual data collection into many scattered sources; lack of knowledge of the best existing health data and covariate sources; economic, legal, and political roadblocks; and heterogeneous timeliness of dataflows. All of these factors affected the time dedicated to this task and the capacity to use data in an efficient way.

First, practitioners encountered difficulties in obtaining complete epidemiological datasets, even for notifiable diseases, due to the heterogeneous quality of epidemiological data provided by physicians, non-exhaustive epidemiological information shared by laboratories, and fragmentation and low interoperability of data sources. PH epidemiologists reported a lack of dedicated databases for epidemiology and a partial digital entry of epidemiological data for notified diseases by medical practitioners. Some data needed for the surveillance of non-notifiable diseases do not exist, as they can be different from those produced for medical care or diagnostic purposes. For instance, practitioners complained about the time-consuming collection of updated complete datasets to monitor endemic diseases, enteric diseases and AMR. Concerning the monitoring of AMR, inadequate timeliness of data collection can be a barrier to producing useful feedback to medical stakeholders and consequently decrease their interest in timely sharing epidemiological data.

*“The problem is that we have very low-quality data from the peripheral labs because they use different systems. They do not truly agree on the list of antimicrobials that should be tested because they have diagnostic needs.”* (Italy, P2, PH)

Trustful sources of validated data describing local sanitary situations were also missing in some countries of interest for Europe (European Neighboring Countries (ENCs) in central Europe and North Africa) and worldwide regarding VBDs (e.g., Zika, chikungunya, dengue and malaria). The time-consuming data collection concerning these countries and diseases was perceived as a barrier to improving travel medicine through more precise risk assessments by location.

Implementing better assessments of the risk of introduction of EID was seen as a challenge in relation to additional dataflows that were missing or time-consuming to collect and merge. Concerning PH, the sources for immunization, medication, travel destinations, and risk behaviors came from one-off studies that were not deemed sufficient. For animal health, information about legal and illegal movements was collected through time-consuming requests to customs or researchers, and there was no centralized source of food products.

Second, EI practitioners had to consult a large number of data sources, and knowledge of the best sources was sometimes person-dependent. The data sources of the EI systems were highly heterogeneous: structured sources, particularly mandatory notification reports, still play a major role in early warning. The EI team of the ECDC stated that they have to consult a high number of structured and unstructured sources, whereas the national officers made strategic choices to reduce the number of unstructured sources in relation to the workload.

EI practitioners complained about manual up-to-date disease data and covariate collection among many scattered sources for VBDs, FWBDs and AMR in PH (national and international scales) but also for epizootics in AH at the international scale. The international primary sources of the most up-to-date validated data were scattered, and their knowledge was often person-dependent. Their identification was based on the practitioner's experience, and there was no alert when a new report or notification of new data became available. This fragmentation and heterogeneity of data producers was also salient at the national level between ministries, medical services, and autonomous regions. This situation led to data sources being left unknown or known but unused.

*“I am sure that there are lots of things that are put officially on certain official ministry websites, with a delay or not. Official data, things that could be useful for us to evaluate epidemiological situations. We do not have access to them because we do not know about them. Then, […] we lose time, so we do not insist.”* (France, AH, P3)

The COVID-19 pandemic increased the workload by overwhelming some notification tools without human moderation or options to receive information limited to diseases of interest. For example, the signals for infectious diseases other than COVID-19 were overshadowed by the increased flow of emails from the EWRS, a tool dedicated to mutual notification for early warning between EU countries.

Third, economic, legal, and political roadblocks hampered data access. Databases were sometimes expensive – for instance, the International Air Transport Association (IATA) data of flights and passengers. Dealing with the regulations for data protection and licensing agreements required specific know-how. For instance, in Italy, the databases used by various institutions (laboratories, hospitals, private doctors), sectors, and administrative units could not be easily merged in 2020 in relation to the new data protection laws. Furthermore, the state authorities sometimes chose not to publish their data or to delay their publication.

*“It is not always possible to find the original source. This is especially true in some countries where the ministry of health is not as transparent as one would want to or the website is not up to date. In some situations, you need to be more imaginative and check with other stakeholders, e.g., MSF, Oxfam, and WHO regional country offices. This is the next step. So, multiple non-direct (*i.e.*, not original) sources would need to be checked.”* (ECDC, P2)

Fourth, the differences in the timeliness of data flows appeared to be a barrier to their integration and analysis needed by stakeholders to implement control measures. The scientific literature was considered an important source of quality but outdated data. The timeliness of IBS dataflows was very heterogeneous according to the sources and diseases: from a one-year delay from city laboratories about AMR to daily reports for COVID-19.

*“If you want to be effective in prevention, you have to start right away, when we detect the problem. [You need] tools that [are] very reactive […]. Today, we are already happy to have an annual temporality of about n*+*1 […]. [But] the PRIMO mission with the city labs, they are able to give, every month, to the labs the analysis that they make of [their data]. If you want to take data from people, you have to offer them something; otherwise, they are not interested!”* (France, PH, P3)

The timeliness of reporting was often considered unsatisfactory for timely data collection. The most prominent European central database repository, TESSy, provided data often seen as outdated for EI practitioners. Concerning international early warning at the European level, the timeliness of IBS dataflows and notifications to international agencies (ECDC, EFSA and WOAH) vary greatly according to the disease. For COVID-19, the health data were notified daily at the national level through dashboards. However, for other infectious threats, such as tick-borne diseases (TBDs), delays in notification and underd-diagnosis did not allow early detection of events.

*“These data are not transferred into TESSy straight away. Therefore, there is always a delay in reporting. In addition, we have to look for information one by one (per member state). We do not do it often because it is so time consuming, only when [it is] something big. […] “Most of the states, not all of them, publish on their websites weekly, and monthly bulletins with the numbers of cases reported for all these diseases. In addition, that is the most up-to-date data that we can access.”* (ECDC, P2)

For some low-income countries, the most up-to-date sources for validated health data were considered to be their ministry websites or social media accounts, but the direct monitoring of social media (not collected by an aggregator) produced a very large amount of data. Facing this difficulty, many practitioners (France and Italy) preferred to monitor only a selection of specific accounts.

#### Data processing and validation

The data processing was seen as a difficult and time-consuming step by the EI practitioners, as EBS and IBS required expert validation.

The formats of the accessible health data or covariates were considered highly heterogeneous because there was no functional and complete informatized platform covering all diseases. PH epidemiologists have reported on the issue of multiple databases in hospital services and laboratories. When databases were available, they were rarely interoperable, especially in EI decentralized systems. When they were not downloadable or not yet available, health data could be found in published reports and bulletins in even less standardized formats. The processing and summarizing of environmental covariates were also seen as a time-consuming effort that was more acceptable in the research setting. The integration of very different data represented a technical barrier for practitioners. In AH, the sources for legal and illegal animal movements and the composition of animal by-products were very different (customs, researchers, scattered nonstructured sources), and their extraction and integration were human-based and very time-consuming.

*“They do not have a standardized system able to collect information for all human diseases, just for some human diseases. In addition, also, many epidemiological investigations, I mean, investigation data that are quite important, they do not have a complete and informatized system.”* (Italy, AH, P3)

The validation of EBS signals was cited as a time-consuming step. When an international event was suspected, it needed to be validated by a committee of experts (in the ECDC, Italy PH and France AH). They had the possibility to contact their networks of focal points (from the ECDC) in Europe and the ENC, reference laboratories, and disease specialists. They also used their personal networks, such as researchers or other professional stakeholders (NGOs). The validation was subject to discussion between peers in the EI teams during daily or weekly briefings, and a consensus could take some time to reach when uncertainty was high. The lack of information exchanges between Eastern European countries rendered the validation of information difficult between national agencies.

The validation of IBS data at the local level was also time-consuming when the number of cases was high and the sources had to be cross-checked, as it was the case for COVID-19 in 2020, which mobilized human resources from other priorities.

“*In addition, when there is a mandatory declaration, there is a whole validation work to be done, which is generally done by the regional health agencies. In addition, this validation work, if there are many, many cases, in fact, hundreds of people would be needed on a permanent basis…*” (France, PH, P4)

Respondents using IBS data stated that they spent time validating them before the risk assessment and field investigation to ensure reliable data even if validation was not their personal duty. The mandatory notification tools and processes were not adapted to diseases with many cases (such as COVID-19), which led to additional work to obtain exhaustive datasets.

*“We know that mandatory reporting does not work when there are too many cases to report.”* (France, PH, P3)

#### Data sharing

The lack of epidemiological data sharing between institutions, sectors and countries was highlighted as a difficulty in relation to structural, technical or political barriers.

Sharing epidemiological datasets between sectors often required a request for special access that is hampered by data protection issues and by the difficulty of managing the very heterogeneous quality of data. Beyond health data sharing, the lack of standardized covariates sharing between sectors and countries was a constraint to implementing standardized intersectoral risk assessments. Building an multisectoral platform was described as challenging and could not cover all diseases.

“*A common database is missing. What happens with veterinarians is not up to them; it is up to the system. That is established in that way. If they do not have a project and sometimes financial support provided, they cannot give us the data because of the Ministry of* Agriculture.” (Serbia, PH, P1)

#### Data analysis

Many EI officers complained about the time-consuming manual work, about a lack of methodological and technical resources to perform sophisticated cross-sectoral analyses concerning AMR, VBDs and FWBDs, and about a lack of quality to share and compare the risk assessments.

First, beyond the time-consuming or technical barriers of data collection and merging, many analyses are still human-based, time-consuming or difficult to implement. EI practitioners complained about the lack of tailored tools used for visualization and automation to analyse large amounts of data and for sophisticated analysis from machine learning, such as for the analysis of social media, identification of AMR genes/new strains or outliers in endemic diseases.

Practitioners highlighted that some knowledge or skills at the scale of the team to perform sophisticated analyses were often missing. There was a lack of consensus about the best intersectoral determinants for the emergence and spread of zoonoses and a lack of expertise in analysing environmental risk. Missing knowledge in epidemiology (e.g., accurate inputs about immune interactions) or missing shared resources (e.g., more complete genomics reference libraries to implement better risk assessments via a One Health approach or access to open-source tools) were cited as important barriers. They stated that they lack the know-how to implement analyses of social media (for early alerts, to monitor trust) and lack knowledge about the available tools.

Practitioners stated that the lack of standardization impacted the quality of risk assessments that can be compared and shared. During the COVID-19 pandemic, the monitoring and comparison of control measures has been challenging, although countries have shared their data through dashboards in a timely manner and relied on the ECDC and research institutes for analysis. The “human covariates” were deemed insufficiently standardized (such as movements and implementation of health measures).

The collaboration between the animal, food, environment and human sectors was considered insufficient by most of the respondents because the coordination from data collection to analysis was lacking: practitioners underlined the lack of standardized shared risk assessments.

We noted an overlap between some mandates of surveillance, with a thin line between the descriptive analysis (situation assessment) and risk assessment for decision-making, making the work more complex for practitioners. Data production by multiple stakeholders can lead to duplication of efforts and difficulty in data merging without strong coordination (e.g., indicators for AMR). It was also challenging to produce analytical outputs adapted to decision-makers. During pandemics (COVID-19 and the HPAI), the production of new modelling outputs was externalized to researchers and specialists. They had to translate their research products into ready-to-use documents for decision-making and communication: taking the criteria of decision-makers into account was necessary to ensure their effective use.

#### Notification and reporting (for the officers in charge)

The difficulties of notification and international reporting were met in relation to the heterogeneity of the IT systems and their lack of interoperability, the quality of the interface, and the reliability of the data for the officers.

The heterogeneity of notification systems was one of the main constraints in countries with a high degree of decentralization, which were organized with autonomous regions and municipalities. In Spain, the lack of homogenization and interoperability was experienced through platforms for national alert networks and national databases of veterinary antibiotic prescriptions (Table 3) that allowed different interpretations across the regions. In addition, large rounds of surveys organized through internal forums may be interpreted differently across regions. A delay in reporting was also identified as a critical problem at the national and subregional levels. When the IT system did not allow automatic uploading of the data, more human resources were needed to fill out the forms online.

Some technical difficulties were also related to constraints on the interface for uploading and frequent changes in the format required for official notification. For example, uploading to TESSy required large datasets to be split into smaller datasets. Furthermore, the frequent changes in the format of data entries induced coordination costs throughout the surveillance systems. The divergences of reporting outputs according to the international system also represented a difficulty for analysis.

*“According to the ECDC system, you have to split your dataset in parts with no more than 500 records. Otherwise, the system is not capable of uploading it. In addition, I have thousands of records. […] [And] It changes quite often. In addition, we have to adapt the system, but […] even a small change in terms of an additional field of data means a big change in the system. Because we have to explain to each lab what the new information means, the way it should be collected, the availability, if it is available or not at their own level. This cannot be done on a one-year basis. If you want new information now, you have to start asking that two years in advance at least.”* (Italy, PH, P2)

At the national level, the problem of reporting could result in a lack of confidence in the reliability of the data for data managers. This problem was also pinpointed in relation to the transparency of data coming from outside European Union that needed careful validation through different networks.

#### Variety of strategic objectives

Strategic stakes linked to different priorities, resources, structural constraints or a lack of exchanges between peers appear to be barriers to analysing data or even organizing surveillance.

The difficulties in implementing multisectoral collaborations highlighted by practitioners were explained by the different prioritization of pathogens for human and animal health, the structural constraints of the institutions and the lack of knowledge about intersectoral relations of causality for emergences (AMR, FWBDs). This was a barrier to building or implementing a One Health plan for AMR despite the political will.

“*Few resources, poor understanding… I think of the problem and at the moment, poor integration between the different parts, the different stakeholders mentioned in that plan*.” (Italy, PH, P2)

Countries had different priorities for IBS of notifiable diseases and different resources to complement it by EBS. They allocated resources to particular diseases depending on the level of risk and control strategy for their country. The interest in *Aedes*-borne diseases was linked to the changing distribution of the mosquito vector species. As such, the risk of introduction and endemization of *Aedes*-borne diseases increased in France. The close monitoring and diagnosis of WNV, which had consequences for blood deferral and vector control, were important in Italy.

When vector control strategies were not sufficient based on the results of entomological monitoring, their direct usefulness decreases, and thus, the quality of their implementation decreased. The lack of cost/benefit analysis of these strategies was a barrier to the revision of these vector control strategies, and the lack of perceived data utility was a barrier to the quality of the vector data collection.

There was a lack of collective reviews of EBS objectives and tools between EI practitioners in general and for AMR in particular. End users expressed the need to have a better prospective approach to build surveillance systems concerning new threats more quickly (this expectation had increased since the Zika epidemic). National officers also expressed the need to exchange experiences and procedures between countries about precise topics such as sentinel monitoring and COVID-19 surveillance.

“*It is more of a problem of staffing and objectives before SOPs. What do we want to see? What do we need to pay attention to? And when it comes to AMR in animals, it is even worse*.” (ECDC, P1)

### Professional logics and propositions for possible improvements

#### Reliance on networks for collective expertise

EI practitioners requested a strengthening of their professional network and exchange channels to reach various objectives: improving the data collection and validation, allowing a continuous review of EI strategies and preparedness between peers and sectors, and facilitating data sharing and consultation.

The mobilization of their professional network was described by EI practitioners as a core feature of their daily work. To carry out their missions, they relied on a large network of epidemiologists in their regional and local administrations, with the addition of private actors or NGOs. These relationships were instrumental in obtaining access to data from supranational organizations (mainly the ECDC, WHO, FAO, WOAH, and EFSA), and collecting a variety of insights and feedback for risk analysis. In AH, multiple collaborations were made with academia, associations of hunters, key stakeholders for wildlife diseases, farmers, and bird protection associations that were part of national sentinel networks.

They also expressed the need for more peer networking between EI practitioners (beyond meetings of focal points organized by the ECDC) to review their EI strategies, objectives, tools and procedures. Collective thinking about priority objectives and feasibility assessments was considered useful for redefining the EBS objectives, in particular for AMR, and thus adapting EBS tools. In a parallel process, the intersectoral list of pathogens produced recently by the EU-JAMRAI project could be reviewed, and comparisons could be made between sectors. Moreover, an optimal set of data requirements for the AH sector (beyond food safety) and environmental sector should be identified.

*“Can we have networks that can help analysts who may come from different backgrounds? To have a comparable evaluation of the information. That is the human component, that is always very present in the surveillance part of the media environment. However, on the other hand, you have got the whole meta-analysis of epidemic intelligence data.”* (Italy, PH, P1)

Some interviewees noted that networking would improve data sharing and consultation among European peers. Epidemiologists performing forecasting for FWBDs would benefit from intercountry feedback as well as AH specialists regarding the use of databases for monitoring antimicrobial use or for building a comprehensive reference genomics repository. In Spain, exchanges of experience were also requested regarding a network of public and private veterinarians performing sentinel surveillance through IT application. Such networks should be strengthened at the European level, as well as at the regional level; the Balkan region has been cited as a relevant perimeter for exchanging official information related to outbreaks.

*“We have gathered some groups of private veterinarians, to see which would be their interest in these applications, how we could focus our approach, these sentinel networks of veterinarians. We have started with dairy herds, and the outcomes are still to be seen. […] It would be very nice a comparison assessment or report about how these things are done in other countries that are participating”* (Spain, AH, P1).

In terms of relationships with decision-makers, preparedness during “peacetime” has been identified as a need. For instance, modelling was used extensively to support decision-making, and a strengthening of the capacities of the modellers for better use of their results by decision-makers has been elicited.

#### More data integration and interoperability

Improving access to data in the multiple dimensions (technical, legal, organizational and political) could be achieved by tools and institutional strategies that vary in terms of centralization and standardization.

First, improved digitization of health data at the level of private physicians and hospitals was still a major expectation. The laboratory dataflows were more digitized and timelier but were not sufficient since the diagnosis of some notifiable diseases relied on clinical or other complementary examinations, as well as the use of paper forms for reporting.

Second, the timeliness and comprehensiveness of national dataflows can be improved, as observed during the COVID-19 pandemic. For example, real-time access to data from sampling laboratories in France was considered to be extended to other diseases. In Italy, the EBS was helpful in bringing context to the case clusters. More complete dataflows could be created thanks to agreements to implement epidemiological objectives in routine or incentive services (by providing quick analytical results as feedback).

“*The example of the laboratory data that comes back on a daily basis, which makes it possible to know how many people have been tested for COVID and how many have tested positive from the sampling laboratories. This raises the question of whether it is sustainable. […] in what form? Will it be anonymous or not? Will it be for all diseases, will it be only for COVID? […] And now we're going to try to set up the same thing for arboviroses as well, using the same pipelines we set up for the COVID, where for the COVID we have direct information from the sampling laboratories*” (France, PH, P1).

Third, a recurrent expectation was to have better data merging and integration to provide complete datasets and to save time for useful analysis. The multiscale integration of data sources in national systems was requested for different epidemiological and administrative databases from different medical services, including pharmacovigilance. This issue could be solved by text mining tools applied to medico-administrative databases, the use of proxies or the effective use of platforms for mandatory diseases (“notification portal”) or OH platforms for enteric pathogens.

“*The reporting portal, where the doctor will enter the information directly online on a single portal. However, that is not just within our jurisdiction; it is also with the Ministry of Health that we need to discuss all this because it will also include pharmacovigilance, vigilance materials, etc., and other vigilances, not just epidemiological surveillance, and with regard to laboratories, there is this whole part of directly recovering activity data from their information system and injecting it directly into the surveillance system*.” (France, P1, PH)

The harmonization and interoperability of infra-national databases could be achieved through a better scientific and institutional concertation of the needed epidemiological datasets (by publishing opinion papers) and a better know-how to manage the new data protection laws. Some issues, AMR in particular, required better coordination between stakeholders to enable indicator collection and integration in both sectors.

*“The more useful data, the minimum amount of data that would be helpful for forecasting and the description of epidemiological situations at the national or international level and the way in which the constraints related to the regulation about data protection… Concerning the exchange of information, the interoperability of the database can be to some extent faced and some solution can be proposed.”* (Italy, PH, P2)

At the international level, several respondents asked for a shared One Health IT system to formalize the OH network and integrate standardized climate, environmental and animal data. EI practitioners requested a better identification of health determinants, particularly for VBDs, and risk thresholds validated by specialists. This identification could be partly done by literature review: it would help to better focus on the most important dataflows to merge. Moreover, it could help agencies to obtain more standardized analyses and allow them to compare their risk assessments. Gathering these data with a OH approach was considered more efficient in terms of decision making. A preference for an interoperable system with the existing platforms was elicited. The general trend was to reduce the number of platforms. User-friendly access to already processed and standardized covariates would allow EI practitioners to save time and to perform more sophisticated intersectoral analyses.

*“All the information in one single place. Therefore, for example, if I see […] that they found anthrax in cattle somewhere in the EU, I want to understand, you know, if that is relevant or not. Therefore, is it the first time? [If not], would it be transmitted to humans at some point, or some other zoonosis? […] There is a platform that deals with zoonotic diseases in animals, another [one] that deals with […] the presence of the disease in humans, [and] the movement of people is on another platform. […] Therefore, to assess which are the relevant [health determinants], we need to jump from one platform to another.”* (ECDC, P2)

Building preselected datasets related to the sanitary context of worldwide locations would help EI officers to perform risk assessments of the introduction of EIDs by travelers (PH). The information about travel locations, mobility and behaviors of travelers could be collected through an app for travelers, social media mining or proxies (TripAdvisor, WTO) and additional dataflows (Eurogate project and covariate repositories).

*“Can you help to centralize or have processed or to store. I found an example: hantavirus in China, which was everywhere in the media last week. I want, for example, to know what is the latest outbreak of Hantavirus in China. Where is this outbreak? How many people live in the cities that are infected? How many people from Europe are travelling to and from this city? What animal reservoir is susceptible to travelling to this city in China? Will I have a mass gathering in this city? All this kind of information.”* (ECDC, P1)

Concerning the risk assessment of introduction by international movements of animals and importation of animal by-products, a European repository could centralize the composition of food products. Another European repository could allow queries of animal and by-product importation and (legal and illegal) movements between countries based on existing dataflows (Eurostat, TRACE database, Movebank and covariates for WNV wildlife, etc.).

The COVID-19 crisis increased the need for EI practitioners to have more knowledge of social and political dimensions for risk analysis as well as communication. Other identified data needs included rumours, context, beliefs and perceptions to monitor trust related to health measures by using sentiment analysis. The possible uses of existing and new tools should be shared among Member States. Complementary tools should also be user-friendly with a manageable quantity of results and settings to monitor trends and visualization and standardized to avoid differences in interpretation between practitioners.

“*You have your indicator-based, you have your EBS, you have a lot of possibilities that you can maintain or reuse, refit as we did for the EBS, so you can put them back when you need them, so it is good to have the instruments in place. In addition, then you can modulate the monitoring response to better suit the situation.*” (Italy, PH, P1)

#### Tools for automation *à la carte*

The need for One Health methodological support and information was recurrent and covered various aspects, from the identification of the best drivers and thresholds of risk for zoonosis (in particular, VBD) to the guidance of preselected open-source tools or semi-automated tools allowing settings and the use of confidential data for visualization or analysis and the use of machine learning to better analyse environmental covariates, genomic data (to detect new strains), and trends for endemic diseases (causal inference and prediction).

A recurring message was that EI practitioners wanted to maintain control of their workflow and their data rather than relying on black boxes. They want to use their own data or/and to choose the data sources. For example, national EI practitioners would like to strengthen their capacities through continuous training, access to R libraries or open-source software and methodological support to choose predefined analytical tools through a decision tree.

They expressed the need for tools that can help them accelerate specific steps on their workflows, depending on the specific characteristics (manual validation or not) of the situations at hand. Although the standardization of the risk assessment was an important objective, the practitioners preferred semi-automation, which allows flexibility of the analyses and a research mindset; for example, a tool that could import and visualize processed covariates and merge to the series of points corresponding to the health/disease data owned by the users would be useful.

*“There is always a physical analyst looking at things, so we have dropped the idea of an artificial intelligence doing everything. However, at the same time, I think automation is enhancing the analyst's job in ways that can better organize the things that you can find, to allow the analyst to have a systematic visualization of things that are together very similar. So you can handle volumes of information more easily.”* (Italy, PH, P1)

One core need of automation was to obtain user-friendly access to centralized information for validated and up-to-date data or EBS data. An alert system indicating the availability of new publications from European laboratories and centres of reference or local bulletins is cited as a useful solution or a tool allowing queries of multiple sources (gathering of international and national validated health data, migration flows and customs for AH, or aggregation of cases published in local bulletins for PH) to assess sanitary situations. The centralization of the alerts and signals in the same formats was seen as important for avoiding the repetition of signals and extracting targeted information.

Machine learning was seen as useful when recurrent complex analyses (such as analysing clusters of bacterial strains) are needed or for detection (trends and outliers in endemic diseases). The production of risk assessments could benefit from analytical tools merging covariates and health data linked to machine learning. For instance, in Italy, respondents would like to integrate environmental covariates and molecular typing of enteric bacterial pathogens to better understand the correlation between outbreaks of FWBDs and the environment and to obtain automatic alerts of new dynamics or abnormalities (causal inferences and predictions).

*“The way the analysis that we are producing now is descriptive only. Some basic reports concerning the number of isolates, trends, and differences in isolation methods according to region, laboratory and pathogen status. We performed several previous studies in which we applied a special analysis to specific outbreaks, and we also included some environmental correlates. In addition, that [gave us] additional information concerning the dynamic, the ecology of some pathogens that were more linked to the environment than others. We would like to implement this kind of analysis.”* (Italy, PH, P2)

User-friendly visualization tools were also mentioned as useful. The building of risk maps to support decision making implies taking into account the criteria of decision-makers (for HPAI, the accurate administrative resolution of the results should avoid stigmatization of farms and allow control measures). The other main use cases included analysing social media and identifying trends, visualizing health data and their covariates, and monitoring outbreaks or endemic diseases in real time. Practitioners would like to have access to settings to choose the analysis period and thresholds of alerts and detection of outliers.

“*It can be much more reworked to maybe even generate buzz level graphs and alerts like that, and that is where it could be improved! Now, we have the raw information that can be tedious to rework manually, but if we set up with macros or with a way of reworking the data that is here in relation to graphs. I think we can go a little bit... We can lighten the information gathering*.” (France, P4, AH)

*“It would be fantastic if we could then apply some formulas that would detect/trigger an alert when there is an increase somewhere and in neighboring countries. For example, a severe increase in scarlet fever, the month before they had 200 cases and the month before that only one case, it is very difficult for a human to [detect] this stuff, unless someone is checking proactively, this could go unnoted, [until] one of the neighboring countries notifies it.”* (ECDC, P2)

The standardization of EBS tools and procedures and better communication about their capacities and roles would help institutions adapt their strategies.

National EI practitioners wanted to be able to adapt their EI strategy and reassess which tasks must be externalized through better information concerning the available tools. Institutional collaboration involved the externalization of complex and time-consuming tasks, such as EBS and modelling, particularly to the ECDC or research institutes. Many countries preferred to use reports or notifications from the ECDC, WOAH and EFSA (Table 3). Specialized EBS tools for early warning, such as social media analysis, were used mainly in dedicated EI teams (the EI team of the ECDC, the Italian network of EI, and the VSI team of the ESA platform), as they were seen as demanding in terms of know-how and continuity of service. In Italy, EBS was used to bring context to the outbreaks of COVID-19 (at the step of community spread) and was seen as very flexible.

“*In Italy, the objective is to support IBS on potential epidemics, or ongoing epidemics in the country. Therefore, we are also interested in information that is normally not very interesting because it is considered normal to have a certain number of infections in the country. In other countries, in other systems that look for what is unusual, what is different from what you expect. Therefore, EBS is very flexible, you can choose your objectives, you can manage it according to what you need in the countries*.” (Italy, PH, P1)

Finally, practitioners expressed the need for a sustainable improvement of practices: they wanted to increase their skills at the scale of the team or institution and replace time-consuming practices in a sustainable way. The maintenance of tools and regular updates of data flows were important issues for ensuring efficient EI systems.

Additional quotes from interviews can be found in Supplementary file 5.

## Discussion

### Diversity of EI systems in the studied European countries

Our study showed that the infrastructures of EI systems and the strategies for early detection and surveillance of EIDs vary across European countries and between the national and regional levels, despite the recommendations of supranational agencies [7]. The organization of surveillance and collaboration differed according to the disease, as stated in a recent cross-sectional study [12]. Despite these differences, all countries in our study benefited from the creation of a health information system (HIS) with different components that are common for many European countries [6]. They combined communicable disease notifications, a sentinel surveillance network with local stakeholders, event-based surveillance, and syndromic surveillance during mass gatherings. The most interoperable and timely databases at the national level were those from laboratories, whose importance for early warning has been recently confirmed by a quantitative study for many models [12]. The shared platforms were useful for intersectoral collaborations, although they cannot be extended to all diseases or health concern. Inside EI, the practices and logics of users showed activities that span from detection to reporting rather than siloed activities for epidemic preparedness and response, as previously described by Barboza [17].

Even if the COVID-19 pandemic allowed more capacity-building in surveillance systems, it increased the workload, tested and strained the existing surveillance systems, and questioned their relevance in contingency time, as previously observed [18]. This observed impact of information overload was also shown in a systematic review of internet-based data for global health surveillance systems [19].

### Use of Event-Based Surveillance

EBS has gained prominence in the past decade. EI practitioners recognized its added value for the timely detection of health threats and its complementarity with IBS, as stated in previous studies [17, 20–24], as well as its insufficient use in decision making [2, 25, 26], as observed for the monitoring of COVID-19. The COVID-19 crisis highlighted the importance of monitoring perceptions and the observance of control measures to enable communication with the public and to identify situations of increased risk. However, media surveillance was sometimes neglected during the COVID-19 pandemic because of decisions to concentrate resources on IBS.

The use of EBS data, in broad meaning (the collection of unstructured data, including nondigital sources, scientific literature and reports of international agencies), was used in all surveillance teams or units, sometimes in a non-regular way. However, the use of specific analytical tools for EBS or rather on the choice of using mainly reports from international agencies was very different between the surveyed countries and depended on the existence of a skilled EI team. Therefore, the situation in 2020 was similar to that in 2006 [27]. Evidence about the performance and acceptability of influenza-like illness (ILI)-specific participatory surveillance systems (InfluenzaNet, Flu Tracking) has been provided, and useful centralization of information and networking (Global Flu View) has been implemented [28]. Some EI teams still preferred to use routine generic platforms centralizing information, such as EIOS, which connects participatory surveillance with one health initiative [29], or ProMED, which is based on a network of local experts reviewing and summarizing health threats [30]. The usefulness of EBS was appreciated in different ways by the EI practitioners in relation to their institutional infrastructure and strategy for early warning and response. Efforts have focused mainly on increasing the timeliness of IBS dataflows, and only some early innovators already used media watches and brought context to their IBS data, even for COVID-19. Other practitioners were focused on international watches (reports from the WHO, Pro-MED, etc.) or even started to explore other EBS sources and methods for analysing social media. Many practitioners have acknowledged the usefulness of text mining tools and application programming interfaces (APIs) applied to structured data such as medico-administrative databases or bulletins to complete epidemiological datasets. This shows that the methods of EBS are useful, go beyond the use of media data, and highlight a continuity between IBS and EBS practices.

However, EBS systems should be vigilant regarding two important challenges, namely, strengthening ethical issues and data protection and ensuring intellectual property protection for researchers and developers [10], and should take advantage of the experience and knowledge of participatory surveillance systems applied to ILI [22, 31].

Our analysis showed the need for more anticipation to integrate readily available EBS tools and skills during interepidemic periods since the capacities of using EBS tools in times of increased workload were linked to the skills developed during peacetime. A stepwise approach is recommended to collectively define the objectives of stakeholders (PH and AH networks) [32]. Better information about the available EBS tools, their functionalities, outcomes and expected workload appears to be a lever to allow agencies to adapt their EI strategies and to take greater advantage of the complementary nature of EBS and IBS.

### Improving the data integration and interoperability

The time-consuming data collection and processing was a priority concern of the practitioners. It translates into a need for centralized access to validated data and already processed covariates, better data integration and interoperability between open and confidential databases. Previous assessments of user needs were based on quantitative surveys, and the needs were investigated in relation to the performances of specific tools [17] or to a preselected solution [33]. Our analysis identified limited knowledge of existing databases and a lack of human resources for extracting and processing data. We confirmed the importance of centralizing data access and descriptive epidemiology in the process of EI [33].

The interviewees linked the difficulties in accessing and processing data to critical technical and organizational barriers. More complete, standardized and timely epidemiological datasets are requested to obtain a more comprehensive view of communicable disease emergence needed to improve prevention. Although some changes are under the regalian responsibility of ministries, some generic and external support can be provided to improve the national dataflows and interoperability of databases. A strengthening of capacities to address the new data protection rules, new knowledge about intersectoral causes of EIDs, identification of the best drivers of EID relevant for different sectors, and scientific networking to obtain agreements about the intersectoral data to collect were identified as useful levers.

Strategies that would allow quicker assessment of risks of introduction include the centralized provision of predefined sets of determinants and health information by disease and location, as well as alerts when new validated datasets are available. These needs expressed by practitioners highlight concrete expectations in line with the general recommendations of connecting data from a wide range of sources [32, 33].

EI practitioners have a cross-border use of IBS and EBS data when gathering data for travel medicine and risk assessment of introduction of EIDs, leading to many challenges in the harmonization of metadata, standardization and ethical frameworks. Lessons learned from platforms such as Global Flu View should be used for the integration of heterogeneous dataflows into repositories or platforms [28].

As notifications and reports from international agencies were used as important data sources, it is important to solve the difficulties impacting the timeliness of reporting by improving the ergonomics of the interface for the upload of datasets and harmonizing the formats of indicators and forms in a sustainable way.

### Strategy and preparedness

More collective thinking and networking between EI peers was suggested by practitioners to solve generic strategic issues related to preparedness (in particular, anticipation of future pandemics) or to the general review of EI strategies and tools and to learn from peers on specific practices (e.g., sentinel surveillance). A recent study highlighted the strong potential for building integrated intersectoral strategies in European neighboring countries concerning VBD detection [34]. New strategic issues arose, such as the review of EBS objectives for AMR and the standardization of social media analysis to monitor the sentiments and behaviors of the general public related to control measures. Our study identified clear expectations for the purposes of networking in line with expert recommendations [25].

### Strengthening the analytical capacities

The interviewees expressed the need for a holistic and integrated approach of surveillance and early warning. The roadblocks to intersectoral collaboration were multidimensional, as observed for AMR. The needs expressed to better implement a One Health approach were also complex. These needs included new epidemiological knowledge about emergencies that concerned different sectors (zoonosis or WFBD), peer networking and collective thinking about surveillance objectives, collective validation of risk thresholds, access to user-friendly tools and methodological support. A multisectoral integrated platform could help intersectoral collaborations by providing standardized resources (such as covariates), generic tools and shared methodological support. This is congruent with a review about the optimization of an integrated OH surveillance system that provided general recommendations beyond EID threats and data management. This highlighted the need to review the intersectoral determinants of EIDs in a broad way (including societal drivers) and to use collaborative disease mapping to develop a more holistic approach to health threats [35].

The question of better use of open-source data and proxies was discussed between EI practitioners and researchers during the workshops on how to produce generic tools that may contribute to harmonized procedures and analysis. This harmonization was an important concern, as was the capacity to select the data sources and the possibility of integrating their own confidential data.

Moreover, the steps in semi-automation must be well characterized by practitioners: sometimes the validation step should remain human-based, but sometimes the collection of already validated data may save time. Precise steps such as the analysis of big data and the production of alerts of outliers should be fully automated to manage large amounts of data; however, EI practitioners wished to maintain control of the final risk assessment. This identified need for semi-automation is more precise than the recommendation of more automation [26]. Solving “basic” needs should be a lever to reach a greater impact than just saving time; for example, user-friendly access to standardized and accurate covariates would allow complex analyses that are not currently implemented, leading to new outcomes. Visualization and machine learning are expected areas of innovation with very different potential outcomes, such as the integration of unstructured data into epidemiological surveillance, causal inferences and predictions. These expectations are in line with a previous qualitative study with artificial intelligence (AI) experts [36] that validated these opportunities and identified the stakes to strengthen the legal framework and collaboration between AI experts and PH/AH practitioners. To increase the acceptability of new methods concerning big data and AI, Zengtao recommended grounding technology developments on practitioners’ problems and guaranteeing the intellectual property of researchers [10].

### Support for the innovation process

This study allows a better understanding of European Member States’ needs and their capacities to implement their own international EI. We observed that if problems were identified, needs were not directly expressed, as they correspond to complex solutions. Some requests, such as reviewing the objectives of surveillance, were sometimes the first phase of the expression of a formal need. Their translation into solutions requires consultation between peers and/or health authorities for strategic issues and discussion with researchers to define technical tools and services and support changes in practices. Our analysis of the practitioners’ discourse allowed us to identify the problems they faced and their associated professional logics. This is required for accurate collective thinking about the paths of solutions that can combine concertation, new knowledge, technical requirements and ergonomics (i.e., mainly a user-friendly interface and procedures that are easy to implement or less time-consuming). The ECDC expressed some proper needs but highlighted their willingness to adapt their practices to Member States’ requests, as past collaboration between the organization and visiting member states showed [20].

The replacement or improvement of practices must be preferred to an additional practice, to help practitioners save time. The sustainability of the tools was a major concern that will condition the changes in practices and thus the ability to reach the stage of innovation. The sustainability of a new platform would need maintenance, updating of the dataflows, flexible use of a panel of generic tools that allows user-friendly settings, methodological guidance and possible use of confidential data. The strengthening of capacities is an important dimension of the innovation process and can rely on building a new expert network [37], summer schools and methodological support.

Agencies were engaged in solving their problems. This is a continuous process in an uncertain world [38–40]. The goal is to identify how to support their innovation process by taking into account what they are already developing and at what stage their thinking and initiatives currently are, as they are sometimes already developing tools with advanced thinking about their deployment and use [41]. We thus have to be careful not to duplicate efforts and innovations.

The prioritization of the needs to address is crucial, and the discussion must involve health authorities concerning issues with data protection, confidentiality and data access, justifying the choice of a cocreation process by the MOOD project. Providing solutions to expectations and needs requires considering constraints specific to national surveillance systems and, at the regional level, the regulatory infrastructure of data access and the context of each country [42]. Several learning loops of interactions are required to increase genericity [43, 44], and the implementation of study cases, as recently recommended by international health authorities [5], is needed to develop a “Global One Health Intelligence System” (GOHIS). A global understanding of practitioners' needs is crucial for the development of feasible, effective, sustainable solutions that can be translated into changes in practices at the national PH and AH scale and feed the European numeric market of open-source tools.

Our findings from this qualitative study can serve as a preliminary basis for an innovation process that fulfills the multidimensional needs of EI practitioners at the national and supranational levels.

### Limitations of this paper

The needs expressed in our manuscript remain person dependent. Some expectations or requests may be connected to the mandate of other national agencies or a particular jurisdiction or administration at the regional level or overseas territories that were not part of our study. Nevertheless, this problem is partially overcome by targeting the leaders and officers involved in risk or situation assessment as gatekeepers of epidemic intelligence activities at the national or European level. In addition, the facilitation workshops, which were organized after the first user need assessment, combined all the stakeholders of the study to discuss particularities and limitations.

Finally, we collected information from users during a period outside the routine activity of surveillance due to the COVID-19 pandemic, influencing their priority needs. The availability of the EI practitioners that were overburdened by the emergency was constrained: we thus targeted the EI team or IBS colleagues to support COVID-19 surveillance rather than specialists in respiratory diseases.

Some answers would have been different outside this period when going into the details, as some procedures have changed, but the main expectations should be quite close. The similarities between AH and PH show that our results are still broadly relevant independent of the period.

### Supplementary Information

**Supplementary Material 1.**

**Supplementary Material 2.** 

**Supplementary Material 3.**

**Supplementary Material 4.**

**Supplementary Material 5.** 

## Data Availability

The analytical data are available in the supplementary files; complementary information can be asked to the corresponding author (neptisliberti@gmail.com). The transcripts generated and analysed during the current study cannot be made available due to the richness of qualitative data and the corresponding risk of reidentification of participants.

## Abbreviations

ADNS: EC's animal disease notification system
AH: Animal health
AMR: Antimicrobial resistance
COVID-19: Coronavirus disease 2019
EBS: Event-based surveillance
ECDC: European centre for disease prevention and control
EFSA: European food safety authority
EI: Epidemic intelligence
EID: Emerging infectious disease
ENC: European neighbouring countries: Algeria, Armenia, Azerbaijan, Belarus, Egypt, Georgia, Israel, Jordan, Lebanon, Libya, Moldova, Morocco, Palestine, Syria, Tunisia and Ukraine
EU-JAMRAI: Joint Action Antimicrobial Resistance and Healthcare-Associated Infections project coordinated by INSERM and ending in 2021
EWRS: The early warning and response system (EWRS) for communicable diseases in the European Union is a web-based system created by the European Commission to "ensure a rapid and effective response by the EU to events (including emergencies) related to communicable diseases
FAO: Food and Agriculture Organization of the United Nations
FTE: Full Time Equivalent
FWBD: Food- and Water-borne Diseases
GOHIS: Global One Health Intelligence System
HIS: Health Information System
HPAI: Highly pathogenic avian influenza
IATA: International Air Transport Association
IBS: Indicator-based surveillance
IHR: International Health Regulation
IT system: Any organized assembly of resources and procedures united and regulated by interaction or interdependence to accomplish a set of specific functions
MoH: Ministry of Health
MOOD: MOnitoring Outbreak events for Disease surveillance in a data science context; https://cordis.europa.eu/project/id/874850
NGO: Non-Governmental Organization
OH: One Health
OIE: Office International des Epizooties (WOAH)
PH: Public Health
RYMY: Ruokamyrkytysepidemiarekisteri (Food Poisoning outbreak register)
SOP: Standard operative procedure
TESSy: The European Surveillance System from ECDC
TRACE: EU system of animal movements and products of animal origin for official certification
VBD: Vector-borne disease
VSI: International Health Surveillance (Veille Sanitaire Internationale)
WHO: World Health Organization
WOAH: World Organization for Animal Health (founded as OIE)
WNV: West Nile Virus
WP: Work Package
WTO: World Trade Organization
