# Supplementary material for: Epidemic intelligence in Europe: a user needs perspective to foster innovation in digital health surveillance
Source: BMC Public Health. 2024 Apr 6;24:973. doi: 10.1186/s12889-024-18466-1 (PMC10999084; doi:10.1186/s12889-024-18466-1)
Supplement: Supplementary file 1 — Supplementary Material 1. [file 12889_2024_18466_MOESM1_ESM.docx]

# Supplementary file 1: Questionnaire for the state of the art in epidemic intelligence

**General information**

Name of the stakeholder:

Name of the interviewee:

Administrative status of the affiliation (national agency/association etc.):

Main mandate of the institution (representation of a sector):
*[The aim of the above question is to have a description of the institute]*

Scope of the stakeholders (public health/animal health/one health)

*Do you have activities with a OH approach? Describe these activities.*

*Are they formalized or situation-specific?*

*If yes, give the definition of a One Health approach*

Which activities have an OH approach and precise if the activities are undertaken at an institute or lower level? (Scale of the OH approach)

Approximate size (number of people) of the institution (affiliation):

**Organization of the EI/disease surveillance staff**

Contact name and role of the person(s) interviewed:

Please describe the EI staff

- Name of service
- Subordination ==> Organization structure + hierarchy (organigramme)
- Number of staff in the team or service

Who is dedicated to EI?

| Institute + department | Name of the person | Roles* | Surveillance system(s)** | Contact information |
| --- | --- | --- | --- | --- |
|  |  |  |  |  |
|  |  |  |  |  |
|  |  |  |  |  |
|  |  |  |  |  |
|  |  |  |  |  |
|  |  |  |  |  |

*Role: Gathering of information

Data management

Data verification and validation

Risk assessment

Reporting (communication)

** Indicator or Event based + WHICH DISEASES?

**General information on the epidemic intelligence/disease surveillance system** * per disease or health concern (such as AMR), where relevant

Country(ies) monitored:
*[The question above is to know whether the institute actively monitoring situation in other countries (than their national territory) if so, which and for what reason?]*

Disease(s) monitored:

- Which are the priority diseases covered by epidemic intelligence?
- Ask about our model diseases if not mentioned *(start with their priority diseases first in order not to bias the answer).*
- **TBE & borreliosis:**
- **West Nile:**
- **Tularemia:**
- **Leptospirosis:**
- **AMR:**
- **Influenza**
- **Disease X**

**For the section below, we start with a general assessment; then, we ask if there is anything different/special for each of the model diseases.**

Do you have a national system for epidemic intelligence/disease surveillance?

Describe the NATIONAL system for epidemic intelligence/disease surveillance

Is it indicator-based or event-based or mixed?

Is it outsourced (ECDC, private companies) or not?

Is it centralized or infra-national/regional?

Who are the main stakeholders involved (Gatekeepers at the department/institute level and main partners/participants)?

- National surveillance?
- International surveillance (if relevant)?
- For national and/or international surveillance?

What is the global work and dataflow (precise the tools if possible) for the EI activities and for each model disease?

Is there a formalized process or is it informal and group-based?

Conclusion...
*Provide legislative references where possible for the mandate of the institution. Provide references for other important stakeholders (linked to epidemic intelligence and surveillance)

**Provide us the organization chart, staff, procedures, manuals for the surveillance (programmes, etc.) at least for the disease case studies
