# Supplementary material for: Epidemic intelligence in Europe: a user needs perspective to foster innovation in digital health surveillance
Source: BMC Public Health. 2024 Apr 6;24:973. doi: 10.1186/s12889-024-18466-1 (PMC10999084; doi:10.1186/s12889-024-18466-1)
Supplement: Supplementary file 2 — Supplementary Material 2. [file 12889_2024_18466_MOESM2_ESM.docx]

# Supplementary file 2. NVivo codes and axes of analysis

| codes (axes in bold) | description |
| --- | --- |
| **Institution, profession** | description of the institution mandate, and attributes of the position of the interviewee |
| mandate of the institution | Mandate and general organization of the institution. |
| professional attributes | Characteristics of the interviewee position that allows to identify his group of peers. |
| **EI data practices** | description of the usual actions concerning the management of Epidemic Intelligence or surveillance data |
| general organization | Main ways to of the manage the work of surveillance and ment of EI data (articulation of through the different steps (with who, how)) |
| collection | practices of management of EI data to collect and filter |
| analysis | management practices of EI data for analysis (aggregation of data, transformation by visualization tools, statistics). |
| information sharing | means any kind of information and data sharing outside the activities of reporting under the EIS (news, knowledges) |
| reporting | activities of official reporting EI data to International agencies (resulting in uploading data in their IT systems) |
| **professional network** | means formal or informal collaboration, could be network of closed or distant colleagues, interdisciplinary activities |
| formal network | description of the structured professional network as organized by the institutional relationships |
| informal network | Description of the noticed collaborations and professional relationships that are not organized by the institutions and rules. |
| One Health | Elements of description of the relationships or collaborations between sectors, initiatives or shared tools in order to take into account the links and dependencies between human health, animal health, plant health, environment. |
| **priority diseases** | means all diseases or health concern addressed by the practitioner |
| Air-borne diseases | means surveillance, research and all activities related to airborne diseases (Flu, Covid mainly) |
| AMR | means surveillance, research and all activities related to bacterial resistances |
| Food and water borne diseases | means surveillance, research and all activities related to food and water borne diseases (including leptospirosis, Tularaemia and AMR concerning the food security) |
| vector-borne diseases | means surveillance, research and all activities related to diseases transmitted by vectors (including WNV, Usutu, Borreliosis (Lyme), TBE) |
| **Problems to solve** | All difficulty, concern, issue expressed by the interviewee (at individual or collective scale) concerning EI or disease surveillance or ways of solving difficulties. |
| difficulties, concerns | any statements of difficulty, concern or problem in relation with the management of EI data/surveillance data |
| solutions | ways/conditions of solving problems expressed in relation with EI data management or surveillance data |
| **useful data and tools** | description of data and tools that are useful for EI or surveillance activities and their characteristics |
| data and tools already used | description of the characteristics of useful data and tools that are currently used |
| wanted data and tools | description of the characteristics of data and tools that must be changed or built. |
| **Impact of covid19** | All statements, practices and logics and Information related to the surveillance of Sars Cov 2/COVID-19 |
| changes (covid) | major or minor new practices in organizational, practices, knowledges and networking due to Sars cov 2/COVID-19 |
| constraints and levers | Issues, in relation to covid-19, opportunities or barriers faced by users that change positively or negatively their way of working and due to covid-19 and ways of solving their problems or minor the constraints |
| Top verbatim | assertions that are eloquent or meaningful. |
