# Supplementary material for: Epidemic intelligence in Europe: a user needs perspective to foster innovation in digital health surveillance
Source: BMC Public Health. 2024 Apr 6;24:973. doi: 10.1186/s12889-024-18466-1 (PMC10999084; doi:10.1186/s12889-024-18466-1)
Supplement: Supplementary file 5 — Supplementary Material 5. [file 12889_2024_18466_MOESM5_ESM.docx]

# Supplementary file 5: Additional quotes from interviews

Detection

*“The early warning and response system has a big problem actually, because of COVID, the amount of information has increased […] immensely. […] If every institute of every country in the EU is responding every day, it means like 20 or 30 replies for a single question and there are several discussions going on there. So, this EWRS notifications, they are filling my email box, right now. There might be something interesting, some Listeriosis for instance, but it is difficult to find because most of the notifications are about COVID.”* (Finland, PH, P2)

Standardization

“*One of the major challenges I would say here and I think applies to many other countries, similar small countries, is that everything is pretty much person dependent. So I tried to when I've been our Team Leader, I've tried to create a lot of documents and so place for different processes that anybody who joins the team can sort of learn how to jump in…*” (Finland, P1, PH)

“*I prefer to maintain this kind of not standardized [workflow]. That is quite flexible for us. At the moment, it is the maximum that I can do with the [human] resources that [we] have. The technical [resources], you can buy, you can develop, you can find. But it's not easy to find, to train and [to keep] skilled people*!” (Italy, P3, AH)

Data merging and integration

*“In general zoonoses… And antimicrobial resistance is another example! The data collected in the two areas, animal health and human health are not compatible. We can't really understand the information from the two sources… We can't really merge them!”* (Italy, P2, PH)

*“Either from the doctor and that will be with what I described to you at the beginning, the reporting portal, where the doctor will enter the information directly online on a single portal. But that's not just within our jurisdiction, it is also with the Ministry of Health that we need to discuss all this, because it will also include pharmacovigilance, vigilance materials, etc, and other vigilances, not just epidemiological surveillance, and with regard to laboratories, there is this whole part of directly recovering activity data from their information system and injecting it directly into the surveillance system.”* (France, P1, PH)

*“The idea is that we need the administrative level of electronic system where any health facility that have a suspicion of any communicable diseases may fill the information about that person, that disease can be in the same time be reported to the district institute and also be visible on national level”* (Serbia, PH, P1)

*“In a hospital, you have a lot of computer systems: you have some in the laboratory, (...), you have bacteriology, but you also have hematology, that's it. There are radiology data, clinical data from the clinical services which are all computerized! And so all this can be put into perspective.”* (France, PH, P4)

Health determinants, One Health approach

*“So I would say that what is called the health determinants are sometimes a factor for us to decide whether there is something to assess, whether something is relevant and if so, how relevant. To make that decision, we may need to jump from one platform to another.”* (ECDC, P1)

*“And then, the point is this difference in quality, but also this very difficult interoperability and the integrated use of those different services, for instance, in animal health and food safety, we have very good data collection systems… Very well centralized… And with quite high data quality inside… From the human side the situation is worse. We have few good data quality, data collection systems… But there is no way to have them analyzed together with the animal health data and the food safety data. So the first concern is the quality. The second concern is interoperability and integration.”* (Italy, P2, PH)

“*We know that there are some areas in which regularly year by year there are some outbreaks of pathogens, water related, mainly environmental related and we think that the water is one of the main sources of exposure in those areas. Then we would like to understand better if there is a straight correlation with environmental covariates… Mainly rainfall and other covariates that can help the understanding of the dynamic in water in order to advise the health authorities in those areas to keep an eye on… In case of environmental conditions that can facilitate the surge of infections*.” (Italy, P2, PH)

*“West Nile virus diseases, that is in Serbia a one health approach. So we are connected with entomologists and veterinarians. Every season, every year we have common programs and common reports. The big reports with them. So one health approach is in a very good level, but not for every disease. Here for this, these are not all diseases on that level.” (*Serbia, PH, P2)

*“We have a lot of informal contact with [public health] authorities, but formally, and there is no constant interexchange. So […] it is difficult for us to get all the information from the human side, and sometimes it is difficult for them to get all the information from our animal health side. I mean, One Health is a challenge for every country at this moment, because there is a lot of inertia from the past opposite of this approach and it is difficult to fight against inertia.”* (Spain, AH, P1)

Analysis

“*We are asked not to carry out a risk assessment because it is not our role, but we are always obliged to do so when we assess an epidemiological situation, so we have done a minimum and this is a cursor that is difficult to place […] In the first stages of the work (writing notes for epidemiological bulletin), one may be limited in what one has to deal with or not to deal with.*” (France, AH, P3).

Automation

“*I do not want to change, I want to improve, I want to improve my method to gain time, gain efficiency in my analysis*.” (ECDC, P1)

“*When we dream of a tool for monitoring. For example, we have an outbreak in a country, say in Poland, with a disease, so we're in France, and if we type in the disease and the species. It would be good to have a database like that that would provide us with all the available information, for example for trade, traced between countries*.*(...)Yes, officially, and also migration*. ” (France, P3, AH)

“*There's always a physical analyst looking at things, so we've dropped the idea that it's artificial intelligence that's doing everything. But at the same time, I think automation is enhancing the analyst's job in ways that can better organise the things that you can find to allow the analyst to have a systematic visualisation of things that are together very similar. So you can, you can handle volumes of information more easily*.” Italy, PH, P1

“*Digested information, like for example, to take the same example, it's Hungary with a case of AI in a farm. So, I type in my software Hungary France, turkey AI. And there, it will take me all the information concerning Hungary from the OIE, from the ADNS, which will take me information on migration. So I won't have to go and look for articles on migration. It's going to look for articles on the website of the Hungarian Ministry of Agriculture and then translate them for me so that I can say here is the information that is available on the website of the Hungarian Ministry of Agriculture. So, I won't have to look for it myself. So here are the channels, the Hungarian professional channels, their website on poultry farming, here is what they have put as information and so everything comes to me already digested.*” (France, AH, P3)

Machine learning

*“If you have different clusters or not. If there are clusters from the time point of view or from the geographical point of view and so on. So it is a completely different analysis that you have to apply here. Here you have to apply a sort of genetic model in clustering models. […] For example, now, what is another field day on which they are working a lot is to study on which, for example, the machine learning approaches. They are developing a lot of them, for the study of the antimicrobial resistance genus and how the the frequency of this genus in the pathogen can be linked with the expression. So the real antimicrobial resistance characteristics that you find of those pathogens.”* (Italy, AH, P3)

Reporting

"*It's really the [member] states that declare after everyone declares in their own way, regularly, in blocks. You have to get to know them. At the beginning, there were also problems between the declarations of the ADNS and the declarations made to the FAO because they were not always consistent. Less and less, there are problems of concordance between the declarations made to the FAO and the ADNS. Now, with the OIE [WOAH], this is not the best way to declare. But there is going to be a new system that will pool declarations between Europe and the OIE [WOAH]. So there will be no more discrepancies.*” (France, AH, P3)
